# Kinematic Evaluation of the GMK Sphere Implant During Gait Activities: A Dynamic Videofluoroscopy Study

**DOI:** 10.1002/jor.24416

**Published:** 2019-08-07

**Authors:** Pascal Schütz, William R. Taylor, Barbara Postolka, Sandro F. Fucentese, Peter P. Koch, Michael A.R. Freeman, Vera Pinskerova, Renate List

**Affiliations:** ^1^ Institute for Biomechanics, D‐HEST ETH Zurich Zurich Switzerland; ^2^ Orthopaedics Balgrist University Hospital Zürich Switzerland; ^3^ Klinik für Orthopädie und Traumatologie Winterthur Cantonal Hospital Winterthur Switzerland; ^4^ The Royal London Hospital London United Kingdom; ^5^ First Orthopaedic Clinic, Faculty of Medicine Charles University Prague Czech Republic

**Keywords:** total knee arthroplasty, medial congruent, moving fluoroscope, single plane fluoroscopy, gait activities

## Abstract

Joint stability is a primary concern in total knee joint replacement. The GMK Sphere prosthesis was specifically designed to provide medial compartment anterior–posterior (A–P) stability, while permitting rotational freedom of the joint through a flat lateral tibial surface. The objective of this study was to establish the changes in joint kinematics introduced by the GMK Sphere prosthesis during gait activities in comparison to conventional posterior‐stabilized (PS) fixed‐bearing and ultra‐congruent (UC) mobile‐bearing geometries. The A–P translation and internal/external rotation of three cohorts, each with 10 good outcome subjects (2.9 ± 1.6 years postop), with a GMK Sphere, GMK PS or GMK UC implant were analysed throughout complete cycles of gait activities using dynamic videofluoroscopy. The GMK Sphere showed the smallest range of medial compartment A–P translation for level walking, downhill walking, and stair descent (3.6 ± 0.9 mm, 3.1 ± 0.8 mm, 3.9 ± 1.3 mm), followed by the GMK UC (5.7 ± 1.0 mm, 8.0 ± 1.7 mm, 8.7 ± 1.9 mm) and the GMK PS (10.3 ± 2.2 mm, 10.1 ± 2.6 mm, 11.6 ± 1.6 mm) geometries. The GMK Sphere exhibited the largest range of lateral compartment A–P translation (12.1 ± 2.2 mm), and the largest range of tibial internal/external rotation (13.2 ± 2.2°), both during stair descent. This study has shown that the GMK Sphere clearly restricts A–P motion of the medial condyle during gait activities while still allowing a large range of axial rotation. The additional comparison against the conventional GMK PS and UC geometries, not only demonstrates that implant geometry is a key factor in governing tibio‐femoral kinematics, but also that the geometry itself probably plays a more dominant role for joint movement than the type of gait activity. © 2019 The Authors. *Journal of Orthopaedic Research* ^®^ published by Wiley Periodicals, Inc. on behalf of Orthopaedic Research Society. J Orthop Res 37:2337–2347, 2019

Mimicking tibio‐femoral kinematics of the healthy knee is thought to be beneficial in the development of total knee arthroplasty (TKA) geometries to maintain sufficient range of motion (ROM) after a total knee replacement, but also not overload the surrounding soft tissue structures. In posterior cruciate retaining as well as in cruciate‐substituting geometries with little conformity between the femoral and tibial articular surface, paradoxical anterior motion has been observed during flexion.^1^ Uncontrolled anterior–posterior (A–P) motion of a TKA could lead to a feeling of joint instability or overloading of the surrounding tissues.^2^, ^3^ A highly constrained geometry is able to restrict A–P condylar motion, but could restrict the functional ROM and raise the required constraining forces, thus promoting implant loosening.^4^

To provide both A–P stability and a large pain‐free range of axial rotation throughout daily activities, medial pivot geometries have recently been introduced. One such geometry, the GMK Sphere prosthesis (Medacta International, Lugano, Switzerland), was specifically designed to constrain the medial condyle through geometrical conformity, while the flat unconstrained lateral tibial surface allows A–P translation of the lateral condyle to permit rotational freedom of the joint. In this respect, the implant is thought to closely mimic the in vitro kinematics of the natural knee.^5^ Until now, the in vivo kinematics of this novel implant geometry have only been investigated during kneeling, lunging, dynamic step‐up/down, and pivoting movements.^6^ Here, little or no translation of the medial femoral condyle was observed, while the lateral condyle translated posteriorly with increasing flexion, resulting in a tibial internal rotation. However, until now, no investigation into the joint kinematics has been undertaken during dynamic gait activities that include functional loading and unloading of the joint, impact at heel‐strike, and changing muscle activation patterns. Since such gait activities belong to the most frequently performed daily tasks, but also challenge subjects with knee disorders,^7^, ^8^, ^9^ their inclusion in a complete evaluation of the functionality of a TKA concept therefore seems critical. In addition, a direct comparison of the in vivo performance of the GMK Sphere to other implant geometries is lacking.

The assessment of joint kinematics has been extensively investigated. In addition to a multitude of examinations using skin marker‐based optical techniques that suffer from errors due to soft tissue artefact,^10^, ^11^, ^12^ imaging techniques such as magnetic resonance imaging,^13^, ^14^, ^15^ roentgen stereophotogrammetry (RSA)^16^, ^17^ and fluoroscopy,^18^, ^19^, ^20^, ^21^ but also bone‐pins^22^, ^23^, ^24^ or cadaveric specimens^5^ were used to provide higher levels of accuracy. Imaging studies have allowed a detailed analysis of the in vivo internal tibio‐femoral kinematics throughout knee flexion, but are generally limited in the examination field of view, and therefore do not allow tracking of the knee joint during full cycles of dynamic gait activities, or are restricted to imaging only a portion of the whole motion. To overcome the constraints of such static imaging approaches, dynamic systems have been developed^25^, ^26^ that now allow investigation into tibio‐femoral kinematics throughout complete cycles of level walking, downhill walking, and stair descent. The use of such a system has, for the first time, recently shown that tibio‐femoral kinematics depend on the activity performed and that clear differences between the loaded stance and the unloaded swing phases of gait activities exist.^27^ Furthermore, it is now known that treadmill walking alters joint kinematics compared with free level walking.^28^

Whether the intended kinematic behavior of the GMK Sphere design principle, that has been presented during lunge and step‐up activities,^6^ is also present for dynamic gait activities and how the kinematics are altered in comparison to conventional posterior‐stabilized (PS) and ultra‐congruent (UC) geometries remains unknown. Therefore, the objective of this study was to compare the in vivo kinematics of the GMK Sphere prosthesis to the conventional GMK Primary PS fixed‐bearing and the GMK Primary UC mobile‐bearing TKA for level walking, downhill walking and stair descent.

## METHODS

### Subjects

In total, 30 subjects with a unilateral TKA and good clinical outcome provided informed written consent to participate in this analytical, observational cohort study (level of evidence 3), which was approved by the local ethics committee (KEK‐ZH‐Nr. 2015‐0140). Three cohorts, each with 10 subjects, possessing either a GMK Sphere (two male/eight female, aged 68.8 ± 9.9, 1.7 ± 0.7 years postop, body mass index [BMI] 25.4 ± 3.7), a GMK Primary PS (5 m/5 f, aged 69.0 ± 6.5, 3.1 ± 1.6 years postop, BMI 27.6 ± 3.5) or a GMK Primary UC (3 m/7 f, aged 75.0 ± 5.1, 3.9 ± 1.5 years postop, BMI 25.9 ± 3.2) implant were measured in the Laboratory for Movement Biomechanics, ETH Zürich, while performing various activities of daily living. Patient selection was performed according to the inclusion criteria (unilateral TKA, ≥1 year postop, BMI ≤ 33, good outcome: WOMAC between 0 and 28 (0–14 excellent, 15–28 good) and pain VAS ≤ 2, good health condition). All the surgical procedures were performed by experienced senior knee surgeons. All TKA surgeries were performed through a medial parapatellar approach with the help of a patient‐specific instrumentation technology (MyKnee; Medacta International, Castel San Pietro, Switzerland). A mechanical alignment (HKA 180 ± 3°) was aimed and no patella was resurfaced.

### Motion Tasks

The kinematics and kinetics of three functional gait activities: level walking (straight ahead on the floor), downhill walking (10° inclined slope), and stair descent (three 0.18 m steps), were captured according to the set‐up described by List et al.^26^ Familiarization trials with the moving fluoroscope (see below) were performed for each activity before acquiring at least five repetitions that included the radiographic assessment.

### Ground Reaction Forces and Motion Capture System

Eight force plates (Kistler AG, Winterthur, Switzerland), which were fully decoupled from the surrounding floor, provided undisturbed ground reaction forces (GRFs) during all measured gait activities.^26^ A GRF threshold of 25 N was used to determine the gait events. The trajectories of a heel marker, captured using an optoelectronic system consisting of 22 infrared cameras (Vicon MX system; Oxfords Metrics Group, Oxford, UK), were used to define the second heel strike event of downhill walking, which was not instrumented with a force plate.

### Moving Fluoroscope

To image complete, consecutive cycles of the knee joint during level walking, downhill walking and stair descent, the moving fluoroscope was employed to capture the relative movements of the femoral and tibial components with a measurement frequency of 25 Hz during the investigated gait activities.^26^, ^27^, ^29^ Detailed information about the videofluoroscopic image capture are provided in the literature.^26^, ^27^, ^29^, ^30^, ^31^, ^32^, ^33^

### Data Processing

#### Two‐dimensional/three‐dimensional (2D/3D) registration

The acquired digital images were corrected for distortion using a local correction algorithm based on a reference grid.^31^, ^34^ The optical projection parameters of the fluoroscopic system, namely focal distance and principal point, were determined from five images of a calibration tube.^31^ The 3D orientation of the implant components was determined using a 2D/3D registration algorithm based on the approach developed by Burckhardt et al.^35^ This process has reported registration errors of ≤0.25° for all rotations, 0.3 mm for in‐plane, and 1.0 mm for out‐of‐plane translations for a similar TKA.^31^, ^34^

### Tibio‐femoral Kinematics

**Figure 1:**
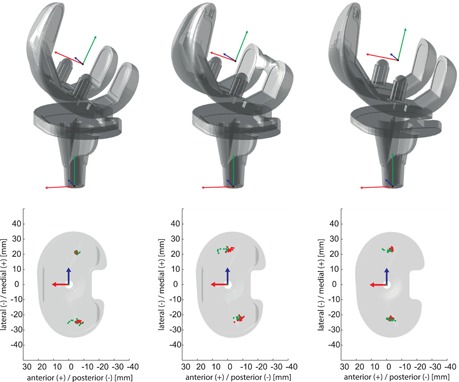
Implant coordinate systems for the femoral and tibial components of the GMK Sphere (left), GMK PS (centre), and GMK UC (right), including the nearest points for stance (red) and swing (green) phases for exemplary trials of level walking presented in the associated coordinate system of the tibial component. [Color figure can be viewed at wileyonlinelibrary.com]

The joint coordinate system approach reported by Grood and Suntay,^36^ based on the femoral and the tibial implant coordinate systems (Fig. 1), has been used in this study to describe the tibio‐femoral rotations. A–P translations of the medial and lateral femoral condyles relative to the top plane of the tibial baseplate were defined using the weighted mean of the ten nearest points on each condyle. To reduce bias due to different implant sizes in the three groups, the locations of the medial and lateral nearest points, presented in the tibial coordinate system, were normalized to a medium femur size with a peg distance of 42.76 mm (normalization factor = 42.76 mm/peg distance). All implant kinematic data were interpolated linearly to allow 101 data points for interpretation over complete activity cycles.

### Statistics

The null hypothesis was defined as no difference in kinematics between the different geometries. To test this hypothesis for the three tibio‐femoral rotations, as well as for the medial and lateral compartment A–P translation of the condyles, five mixed‐model analysis of variances (ANOVAs) with subject as a random effect were performed. Here, the influence of the geometry was investigated with rotational (flexion/extension, internal/external, and ab/adduction) and translational (medial compartment A–P, lateral compartment A–P) ROMs during complete cycles, as dependent variables, and geometry with three levels (GMK Sphere, GMK PS, and GMK UC) and task with three levels (level walking, downhill walking, and stair descent) as the independent variables. Post hoc comparisons were conducted using a least significant differences (LSD) approach and significance levels were adjusted for multiple comparisons using Bonferroni correction. All ANOVAs were conducted in SPSS (SPSS v24; IBM, Armonk, NY).

## RESULTS

**Figure 2:**
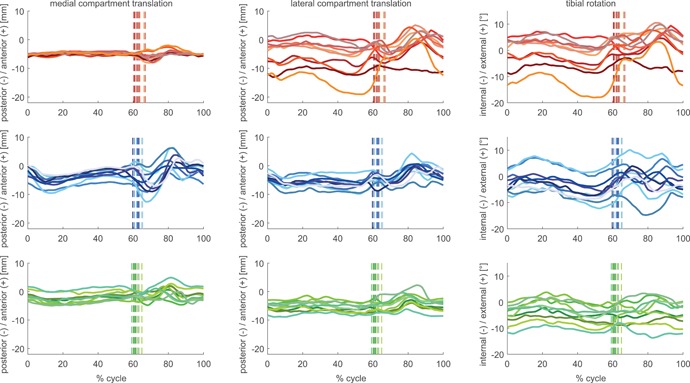
Subject means of condylar anterior–posterior (A–P) translation and tibial rotation for the GMK Sphere (red tones), GMK PS (blue tones), and GMK UC (green tones) throughout full cycles of level walking. The average instance of toe‐off of each subject is shown as a vertical line. [Color figure can be viewed at wileyonlinelibrary.com]

**Figure 3:**
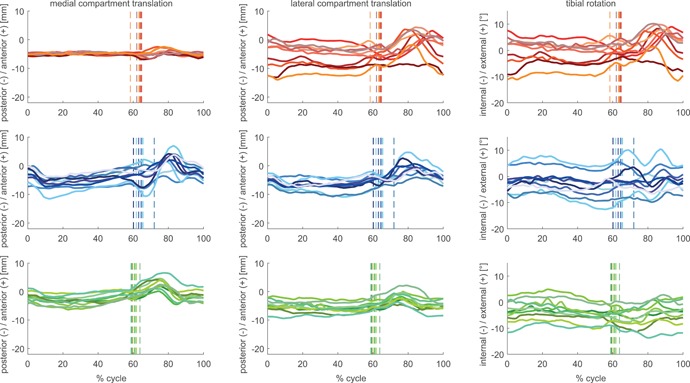
Subject means of condylar anterior–posterior (A–P) translation and tibial rotation for the GMK Sphere (red tones), GMK PS (blue tones), and GMK UC (green tones) throughout full cycles of downhill walking. The average instance of toe‐off for each subject is shown as a vertical line. [Color figure can be viewed at wileyonlinelibrary.com]

**Figure 4:**
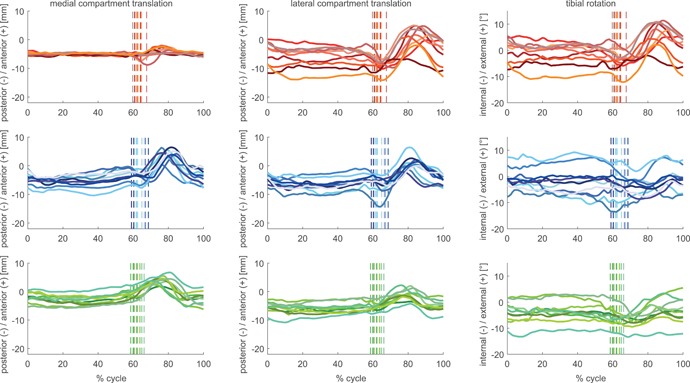
Subject means of condylar anterior–posterior (A–P) translation and tibial rotation for the GMK Sphere (red tones), GMK PS (blue tones), and GMK UC (green tones) throughout full cycles of stair descent. The average instance of toe‐off for each subject is shown as a vertical line. [Color figure can be viewed at wileyonlinelibrary.com]

**Figure 5:**
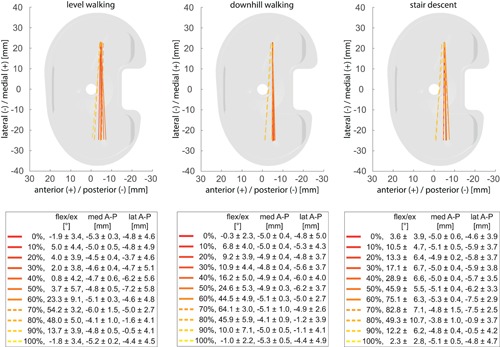
Average positions of the femoral component of the GMK Sphere, represented by lines connecting the nearest points of the medial and lateral condyles relative to the tibial tray for specific time points during the gait cycles of the three activities. Solid lines represent the loaded stance phase and dotted lines the unloaded swing phase. Mean and standard deviation of flexion/extension (flex/ex) as well as the anterior–posterior (A–P) translation of the medial (med) and lateral (lat) condyles over the subject group for the selected time points are also presented. [Color figure can be viewed at wileyonlinelibrary.com]

**Figure 6:**
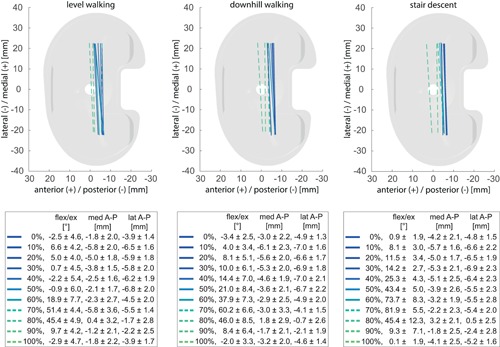
Average positions of the femoral component of the GMK PS, represented by lines connecting the nearest points of the medial and lateral condyles relative to the tibial tray for specific time points during the gait cycles of the three activities. Solid lines represent the loaded stance phase and dotted lines the unloaded swing phase. Mean and standard deviation of flexion/extension (flex/ex) as well as the A–P translation of the medial (med) and lateral (lat) condyles over the subject group for the selected time points are also presented. [Color figure can be viewed at wileyonlinelibrary.com]

**Figure 7:**
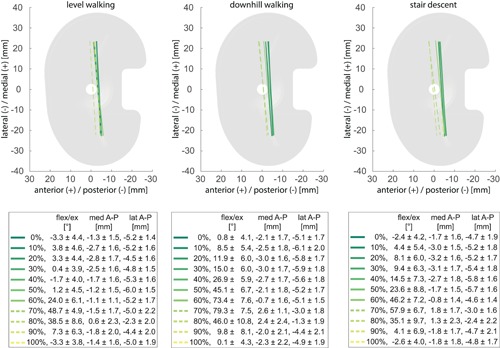
Average positions of the femoral component of the GMK UC, represented by lines connecting the nearest points of the medial and lateral condyles relative to the tibial tray for specific time points during the gait cycles of the three activities. Solid lines represent the loaded stance phase and dotted lines the unloaded swing phase. Mean and standard deviation of flexion/extension (flex/ex) as well as the anterior–posterior (A–P) translation of the medial (med) and lateral (lat) condyles over the subject group for the selected time points are also presented. [Color figure can be viewed at wileyonlinelibrary.com]

In general, the implant geometry influenced the kinematic patterns to a greater degree than the different gait activities (Figs. 2–7). While a low level of inter‐subject variability was observed on the medial condyle of the GMK Sphere, the greatest variability in tibio‐femoral kinematics was observed on its lateral side.

### Rotations

All three implants exhibited equal ranges of joint flexion during downhill walking and stair descent, but the GMK Primary UC showed significantly reduced flexion during level walking compared with the GMK Sphere and GMK PS (Table 1). The GMK Sphere exhibited a significantly larger range of tibial internal/external rotation compared with the GMK UC for all tasks and compared with the GMK PS for stair descent. No differences were found between the implant geometry kinematics for ab/adduction.

**Table 1:** Range of Motions for all Three Tibio‐Femoral Rotations for the GMK Sphere, GMK PS, and GMK UC During Level Walking, Downhill Walking, and Stair Descent

	Complete Gait Cycle			Loaded Stance Phase			Unloaded Swing Phase		
	flex/ex [°]	int/ext [°]	ab/add [°]	flex/ex [°]	int/ext [°]	ab/add [°]	flex/ex [°]	int/ext [°]	ab/add [°]
Level walking									
GMK Sphere	62.7 ± 4.9^*^ ^a^	11.9 ± 4.2^*^ ^c^	2.8 ± 0.8	34.2 ± 6.2	7.3 ± 2.8	1.8 ± 0.4	61.9 ± 5.4	9.2 ± 3.2	2.5 ± 0.7
GMK PS	63.5 ± 4.7^*^ ^b^	10.5 ± 1.9	2.9 ± 0.8	33.0 ± 7.2	7.9 ± 1.3	2.2 ± 0.6	61.8 ± 5.7	8.3 ± 1.5	2.3 ± 0.7
GMK UC	57.2 ± 4.8^*^ ^a,b^	8.1 ± 2.5^*^ ^c^	2.3 ± 0.6	30.2 ± 4.2	6.2 ± 2.8	1.6 ± 0.3	56.4 ± 5.5	5.9 ± 1.6	2.0 ± 0.7
Downhill									
GMK Sphere	70.0 ± 4.5	11.5 ± 2.7^*^ ^d^	2.6 ± 1.0	51.5 ± 6.0	6.3 ± 1.7	1.5 ± 0.2	69.9 ± 4.6	10.1 ± 2.5	2.3 ± 0.9
GMK PS	69.9 ± 5.3	8.9 ± 2.1	2.6 ± 0.7	50.1 ± 8.3	6.3 ± 2.0	2.1 ± 0.7	68.7 ± 6.3	7.3 ± 1.8	2.0 ± 0.6
GMK UC	66.1 ± 3.4	7.9 ± 2.3^*^ ^d^	2.2 ± 0.7	47.3 ± 4.8	5.6 ± 1.9	1.5 ± 0.2	65.6 ± 3.8	6.1 ± 1.9	1.9 ± 0.7
Stair descent									
GMK Sphere	89.5 ± 5.5	13.2 ± 2.2^*^ ^e,f^	3.0 ± 1.2	77.5 ± 6.4	6.6 ± 2.4	2.1 ± 0.8	89.1 ± 5.3	12.8 ± 2.4	2.3 ± 1.0
GMK PS	90.2 ± 5.5	9.0 ± 2.5^*^ ^e^	2.9 ± 0.7	78.8 ± 9.2	6.6 ± 3.1	2.5 ± 0.7	89.9 ± 5.6	7.6 ± 1.9	2.0 ± 0.6
GMK UC	87.5 ± 4.4	8.4 ± 3.3^*^ ^f^	2.3 ± 0.7	76.6 ± 5.6	6.0 ± 2.5	2.0 ± 0.5	87.3 ± 4.5	7.3 ± 3.3	1.7 ± 0.5

Mean and standard deviation for all groups are reported for: flexion/extension (flex/ex), tibial internal/external rotation (int/ext), and abduction/adduction (ab/add). Complete gait cycles as well as loaded stance and swing phase are presented.

^*^ Significant differences (a–f), based on the adjusted level of significance of α = 0.0056.

**Figure 8:**
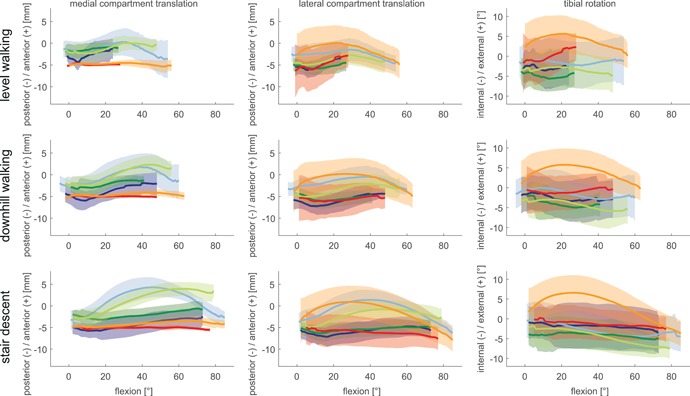
A–P translation of the medial (left) and lateral (centre) condyle as well as tibial rotations (right) for specific flexion angles. Mean and standard deviations over the subject groups are presented for the GMK Sphere (red/orange), GMK Primary PS (blue/light blue), and GMK Primary UC (green/light green) for the loaded stance (dark) and unloaded swing (light) phases of level walking, downhill walking, and stair descent. [Color figure can be viewed at wileyonlinelibrary.com]

The tibial components of the GMK PS and UC geometries remained, on average, internally rotated throughout the full range of flexion of all activities, including loaded stance and unloaded swing phases (Fig. 8). In contrast, the GMK Sphere exhibited a different kinematic coupling between flexion and tibial rotation especially for the loaded stance phase of level walking and the unloaded swing phases of all activities. Here, the GMK Sphere showed an increase in external rotation, which was followed by an internal rotation, but the orientation of the tibia relative to the femoral component generally remained externally rotated. No clear differences in tibial rotation could be seen for the loaded stance phases of stair descent and downhill walking compared with the conventional geometries.

### A–P‐Translation

The motion patterns of the mean A–P translations of each subject (Figs. 2–4) for the GMK Sphere showed very constrained medial condylar motion with almost no inter‐subject variation throughout full cycles of level walking (mean SD 0.6 mm), downhill walking (mean SD 0.5 mm), and stair descent (mean SD 0.6 mm), whereas the lateral condyle was found to allow subject‐specific motion patterns, resulting in high inter‐subject variability (mean SD level walking 4.6 mm, downhill 3.8 mm, stair descent 3.6 mm). In contrast, the two conventional geometries exhibited similar inter‐subject variability for both condyles with slightly larger variation for the GMK PS (mean SD medial 2.2–2.4 mm, lateral 1.9–2.3 mm), than for the GMK UC geometry (mean SD medial 1.7–1.8 mm, lateral 1.8–1.8 mm). Of importance was that intra‐subject variability was extremely low (maximal mean SD for a single subject was 1.5 mm, observed during stair descent).

The medial compartment range of A–P translation differed significantly between all three investigated geometries (Table 2). Here, GMK Sphere showed the smallest A–P translation for the medial condyle for level walking, downhill walking, and stair descent, followed by the GMK UC and the GMK PS (Figs. 5–7). For the lateral condyle, the GMK UC showed a significantly smaller range of A–P translation for all activities compared with the GMK Sphere and to the GMK PS. For all geometries, the ranges of A–P translation for both condyles were smaller during the loaded stance phases compared with the unloaded swing phases (Figs. 2–7, Table 2), with minimal mean ranges found during stance for the medial condyle of the GMK Sphere of 2.0 ± 0.2 mm for level walking, downhill walking 1.7 ± 03 mm, and stair descent 2.4 ± 0.9 mm.

**Table 2:** Range of A–P Translation for the Medial (med) and Lateral (lat) Condyles for the GMK Sphere, GMK PS, and GMK UC During Level Walking, Downhill Walking and Stair Descent

	Complete Gait Cycle		Loaded Stance Phase		Unloaded Swing Phase	
	Med A–P	Lat A–P	Med A–P	Lat A–P	Med A–P	Lat A–P
Level walking							
GMK Sphere	3.6 ± 0.9^*^ ^a,b^	10.6 ± 4.4^*^ ^d^	2.0 ± 0.2	6.1 ± 2.8	3.3 ± 1.1	8.5 ± 2.4
GMK PS	10.3 ± 2.2^*^ ^a,c^	8.4 ± 1.6^*^ ^e^	5.7 ± 1.1	5.0 ± 1.0	9.5 ± 2.4	6.9 ± 2.0
GMK UC	5.7 ± 1.0^*^ ^b,c^	5.5 ± 1.4^*^ ^d,e^	3.3 ± 1.2	3.4 ± 1.2	4.9 ± 0.8	4.8 ± 1.1
Downhill						
GMK Sphere	3.1 ± 0.8^*^ ^f,g^	9.9 ± 3.0^*^ ^i^	1.7 ± 0.3	5.1 ± 1.3	2.9 ± 1.0	8.7 ± 2.4
GMK PS	10.1 ± 2.6^*^ ^f,h^	9.1 ± 2.3^*^ ^j^	5.3 ± 1.6	4.9 ± 1.1	8.3 ± 2.9	6.4 ± 1.9
GMK UC	8.0 ± 1.7^*^ ^g,h^	5.7 ± 1.1^*^ ^i,j^	3.8 ± 0.8	3.0 ± 1.0	6.6 ± 1.3	4.8 ± 1.2
Stair descent						
GMK Sphere	3.9 ± 1.3^*^ ^k,l^	12.1 ± 2.2^*^ ^n^	2.4 ± 0.9	5.6 ± 2.2	3.4 ± 1.6	11.9 ± 2.3
GMK PS	11.6 ± 1.6^*^ ^k,m^	10.7 ± 2.6^*^ ^o^	4.6 ± 1.2	5.1 ± 1.2	10.2 ± 1.6	9.7 ± 2.6
GMK UC	8.7 ± 1.9^*^ ^l,m^	6.9 ± 1.6^*^ ^n,o^	4.2 ± 1.1	3.6 ± 0.9	7.7 ± 1.9	5.7 ± 1.4

Mean and standard deviation for all groups during complete gait cycles as well as loaded stance and swing phase are presented.

^*^ Significant differences (a–o), based on the adjusted level of significance of α = 0.0056.

The largest and smallest range of A–P translation found throughout full cycles of the activities in a single trial were observed in a medial (1.5 mm during downhill walking) and lateral (20.7 mm during level walking) condyle of two subjects with a GMK Sphere implant.

The conventional geometries exhibited similar kinematic coupling characteristics (relationship between joint flexion, A–P translation, and internal/external rotation) for the medial and lateral condyles, but with considerable differences between the loaded stance and unloaded swing phases (Fig. 8). The medial condyle of the GMK Sphere exhibited almost no translation over the full range of joint flexion for all activities and even for the unloaded phases. The lateral condyle, however, exhibited a kinematic coupling comparable to the conventional geometries but with a large variation between subjects. Posterior translation with increasing flexion was found for the lateral condyle of the GMK Sphere during the loaded stance phase of stair descent. However, different patterns of tibio‐femoral movement with joint flexion were found for the conventional PS and UC geometries as well as the lateral condyle of the GMK Sphere for the stance phase of level walking and downhill walking. Here, little or only anterior translation was observed for flexion angles >15–20°. In general (apart from the medial condyle of the GMK Sphere) all condyles then exhibited a greater ROM, moving anteriorly with a different kinematic coupling pattern, for all unloaded swing phases.

## DISCUSSION

The sphere‐in‐sphere articulation on the medial side of the GMK Sphere implant is a geometry characteristic that was included to provide A–P stability of the replacement joint. The flat lateral condyle was then intended to allow rotational freedom, with the ultimate goal of mimicking the kinematics of the healthy knee joint.^5^ While a preliminary understanding of the effectiveness of this geometry has been provided in subjects undertaking step‐up/down,^6^ for the first time, the in vivo kinematics of the GMK Sphere prosthesis have now been analysed during level walking, downhill walking and stair descent. These measurements have been enabled by the unique ability of the moving fluoroscope^26^ that has allowed the assessment of 3D tibio‐femoral kinematics throughout complete cycles of different gait activities without errors being introduced due to soft tissue artefact.^10^, ^24^ The additional comparison against conventional GMK PS and UC geometries using the same methodology, not only demonstrates that implant geometry is a key factor in driving tibio‐femoral kinematics, but also that the geometry itself can play a more dominant role for joint movement than the type of activity being undertaken.

The mean ranges of tibio‐femoral rotation (Table 1) of the GMK Sphere over the complete gait cycles were equal or even larger than the PS and UC geometries in all three planes and for all activities, suggesting that little or no additional restrictions in axial rotation range occur due to the medially constrained compartment provided the lateral compartment is unconstrained. The lower range of rotation in the UC geometry, however, indicates that the implant with constrained medial and lateral compartments may restrict the joint axial rotation. Here, the UC geometry shows comparable values to those reported for level walking and stair descent with a cruciate retaining implant.^27^ The ranges of tibial rotation determined during the loaded stance phase of level walking (GMK Sphere: 7.3 ± 2.8°, GMK PS: 7.9 ± 1.3, GMK UC: 6.2 ± 2.8) and stair descent (GMK Sphere: 6.6 ± 2.4°, GMK PS: 6.6 ± 3.1, GMK UC: 6.0 ± 2.5) were comparable to studies investigating the healthy knee in vivo during the stance phase of normal gait and stair descent.^18^, ^22^, ^24^, ^37^ The largest range of tibial rotation, however, was clearly observed during the unloaded swing phase of gait, which is consistent with the study of natural knee kinematics by Lafortune et al.^22^

The results of our study demonstrate that a distinct kinematic coupling exists between the average tibial‐rotation and the joint flexion angle for the GMK Sphere for the unloaded swing phases of all activities (also the stance phase of level walking). The observed externally rotated tibia indicates that the specific geometry of the GMK Sphere leads to more external rotation (depending on the loading and the activity) than the more conventional geometries. When comparing the joint kinematics during the loaded stance phase of stair descent against the results of a dynamic step‐up/down activity investigated by Scott et al.,^6^ who found a general increase in internal tibial‐rotation with increasing flexion, the small increase in internal rotation with increasing flexion angle observed in our study (Fig. 8), does indeed suggest that a kinematic coupling occurs. For the additional activities investigated in our study, for example, the stance phase of level walking, however, a contrary coupling was observed, with increasing tibial external rotation with flexion, indicating that tibial rotation has a relationship with flexion angle that also varies with implant geometry and activity.^22^, ^27^ In both cases, it should be noted that large inter‐subject variations were observed, and interpretation of the “average” kinematic coupling needs to be interpreted carefully.

The high repeatability in A–P translation between the five intra‐subject trials allows subject‐specific motion characteristics to be distinguished (Figs. 2–4), indicating that less constraints imposed by the implant geometry results in highly individual motion characteristics. The highly constrained medial side of the GMK Sphere exhibited the lowest inter‐subject variation (average SD 0.5–0.6 mm), and the subject‐specific motion characteristics therefore appear to be almost exclusively expressed on the minimally constrained lateral side of the implant (average SD 3.6–4.6 mm). The slightly larger variation found for the PS implant for both condyles (average SD 1.9–2.4 mm) compared with the UC geometry (average SD 1.7–1.8 mm) confirms the influence of the component geometry constraints. The individual motion characteristics found within the implant groups might additionally be explained by other factors including anatomy, component alignment, ligament tension, muscle activation, or individual spatial/temporal gait characteristics, but the relative influence of these factors on joint kinematics remains to be investigated in further studies.

Interestingly, for all activities, subject‐specific A–P translation in the highly constrained medial sphere‐in‐sphere articulation of the GMK Sphere implant occurs only at the beginning of swing phase (Figs. 2–4). This increase in A–P translation seems to have been made possible by the spherical condyle lifting‐off out of the socket after unloading the joint and before the contraction of the surrounding muscles of the knee preparing for the on‐coming heel‐strike. An increase in proximo‐distal distance between the femoral and tibial component was also observed at the same time and in the same subjects. Whether this kinematic phenomenon results from the femoral component rolling or sliding up the inlay, or whether it is associated with either joint abduction or a collateral ligament laxity induced (uni‐ or bi‐lateral) lift‐off remains to be investigated. However, this finding seems to underline the importance of ligament balancing on the in vivo kinematics of the GMK Sphere during unloaded phases of gait activities.

The significantly smaller ranges of A–P translation found for the medial condyle of the GMK Sphere (stance: 1.7–2.4 mm, swing: 2.9–3.4 mm) during the three gait activities, as compared with the conventional geometries, indicates a high level of A–P restriction for the medial condyle. The ranges of A–P translation of the lateral condyle (stance: 5.1–6.1 mm, swing: 8.5–11.9 mm) did not differ from the ranges found for the PS geometry, and were larger than those exhibited by the UC geometry. Similar to the values determined for joint rotation, these values suggest that the restricted medial condyle of the GMK Sphere does not limit the ROM of the lateral condyle. These ROMs for the medial and lateral condyles of the GMK Sphere were comparable to the A–P translations found for the healthy knee during loaded stance phases of level walking and stair descent,^18^, ^38^ but smaller in magnitude for the lateral condyle compared with deep knee bend or squatting activities that include larger flexion angles.^15^, ^39^ A comparison of these kinematic results against A–P translations for natural knees during gait activities remains extremely difficult, primarily because healthy knee kinematics themselves remain controversially discussed,^18^, ^37^ but also due to the low numbers of subjects involved in the described studies.

Posterior translation of the lateral condyle with increasing flexion, as described by Iwaki et al.^5^ for cadaveric knees and found for the GMK Sphere during lunge and step‐up/down activities by Scott et al.,^6^ was for the loaded stance phases only seen during stair descent of the GMK Sphere (Fig. 8, centre). The so‐called “paradoxical” movement reported in the literature,^1^ describes anterior translation of the femoral condyle(s), despite increasing joint flexion. In our study, the loaded stance phases of the conventional PS and UC implants for both condyles during all activities, showed tendencies toward anterior translation (at flexion angles >15–20°), thus confirming the presence of this paradoxical type motion. It is interesting to note that femoral anterior movement was also observed for the lateral condyle of the GMK Sphere during the late stance phase of level walking, when increasing flexion was combined with unloading—the first occurrence of this observation in this implant. The distinct kinematic behavior of the three gait activities and loaded and unloaded phases highlights the importance of including different gait activities for an improved evaluation of implant geometry.

It must be noted that a number of factors limit the extrapolation of the findings of this study for a general understanding of joint biomechanics in larger populations. Here, the low number of subjects examined, combined with an unequal gender distribution and an older age of the UC patients, could bias the observed kinematic outcomes, and thereby restrict a comprehensive understanding of the differences in motion characteristics between implants. While additional subjects could elucidate the extreme ROM, some of the differences in subject‐specific kinematics are already clear even in the low number of subjects examined in this study, and already highlight the condylar ROM freedom and activity dependency within the geometry specific constraints. Since osteoarthritis and following TKA is more frequent in female patients, the patient selection did not aim in achieving an equal gender distribution and any influences of gender dependency on knee kinematics would need to be addressed in future studies. The low gait speeds of the subjects walking with the moving fluoroscope has been addressed previously,^40^ in which it is already known that the kinematics resemble those during slow walking. From an analysis perspective, the out of plane error of the single plane fluoroscope^26^ only allows a limited evaluation of the medio‐lateral translation of the implant components, which have therefore not been reported in this study. However, the use of this technology has clearly allowed a new understanding of implant kinematics throughout complete cycles of functional gait activities, including the effects of muscle contraction and relaxation, as well as loaded and unloaded activity phases.

The results of this study confirm that the design principle of the GMK Sphere, in providing limited medial compartment A–P ROM through a medial sphere‐in‐sphere articulation and allowing large ranges of axial rotation through a flat lateral compartment, is successful in terms of the in vivo kinematics produced throughout the performed gait activities. From a clinical perspective, whether patients prefer the medial stabilized implant over conventional geometries^3^ and the effect of these geometries on constraining forces, was not the focus of this study, and remains to be assessed elsewhere. Furthermore, the threshold of A–P translation that distinguishes between stability and instability cannot be defined on the present data of good outcome subjects, but should be part of further studies looking at bad outcome subjects and a possible correlation between a feeling of instability and range of A–P motion. An improved knowledge of healthy tibio‐femoral kinematics during similar complete cycles of dynamic functional gait activities, as well as their modulating factors, remains critically required before implant geometries are better able to mimic healthy joint motion. In this study, however, we have been able to show that innovative TKA geometries such as the GMK Sphere are able to restrict medial condylar A–P motion and still allow large ranges of axial rotation, but that the motion of the lateral condyle is still highly subject‐specific and activity dependent.

## AUTHORS’ CONTRIBUTION

P.S., S.F., and P.K. recruited the subjects; P.S., B.P., and R.L. performed the measurements; W.T., M.F., S.F., P.K., V.P., R.L., and P.S. were involved in planning and design of the study; R.L., W.T., and P.S. supervised the work; P.S. and B.P. processed the experimental data; P.S., B.P., and R.L. performed the analysis; P.S., W.T., R.L., S.F., P.K., M.F., and V.P. aided in interpreting the results; P.S. and R.L. drafted the manuscript and designed the figures; W.T. and R.L. provided the resources, and worked on the manuscript. All authors discussed the results and commented on the manuscript. All authors gave final approval for publication.
